# High Precisive Prediction of Aflatoxin B_1_ in Pressing Peanut Oil Using Raman Spectra Combined with Multivariate Data Analysis

**DOI:** 10.3390/foods11111565

**Published:** 2022-05-26

**Authors:** Chengyun Zhu, Hui Jiang, Quansheng Chen

**Affiliations:** 1 School of Physics and Electronic Engineering, Yancheng Teachers University, Yancheng 224007, China; zhucy@yctu.edu.cn; 2 Jiangsu Intelligent Optoelectronic Devices and Measurement and Control Engineering Research Center, Yancheng 224007, China; 3 School of Electrical and Information Engineering, Jiangsu University, Zhenjiang 212013, China; 4 School of Food and Biological Engineering, Jiangsu University, Zhenjiang 212013, China; qschen@ujs.edu.cn

**Keywords:** peanut oil, aflatoxin B_1_, Raman spectroscopy, characteristic wavelength optimization, partial least squares

## Abstract

This study proposes a label-free rapid detection method for aflatoxin B_1_ (AFB_1_) in pressing peanut oil based on Raman spectroscopy technology combined with appropriate chemometric methods. A DXR laser Raman spectrometer was used to acquire the Raman spectra of the pressed peanut oil samples, and the obtained spectra were preprocessed by wavelet transform (WT) combined with adaptive iteratively reweighted penalized least squares (airPLS). The competitive adaptive reweighted sampling (CARS) method was used to optimize the characteristic bands of the Raman spectra pretreated by the WT + airPLS, and a partial least squares (PLS) detection model for the AFB_1_ content was established based on the features optimized. The results obtained showed that the root mean square error of prediction (RMSEP) and determination coefficient of prediction ($R_{P}^{2}$) of the optimal CARS-PLS model in the prediction set were 22.6 µg/kg and 0.99, respectively. The results demonstrate that the Raman spectroscopy combined with appropriate chemometrics can be used to quickly detect the safety of edible oil with high precision. The overall results can provide a technical basis and method reference for the design and development of the portable Raman spectroscopy system for the quality and safety detection of edible oil storage, and also provide a green tool for fast on-site analysis for regulatory authorities of edible oil and production enterprises of edible oil.

## 1. Introduction

Aflatoxins are the metabolites of Aspergillus flavus and Aspergillus parasiticus, and exist in moldy grains, oil plants and foods made from grains and oil plants [1]. There are various isomers of aflatoxin, among which aflatoxin B_1_ (AFB_1_) is the most toxic and carcinogenic, and its acute toxicity is 10 times that of potassium cyanide, which can cause liver, stomach, and kidney cancer in humans and animals [2]. In 1993, The AFB_1_ was classified as a Group I carcinogen by the International Agency for Research on Cancer (IARC). The chemical structure of the AFB_1_ is very stable and can only be decomposed when heated to 268 °C, and it is difficult to eliminate it by ordinary food processing methods [3]. Once the AFB_1_ enters the human body, it causes great harm to human health [4]. Under normal circumstances, edible oils rarely contain aflatoxins. When the oil plants are not stored properly, mildew will occur, resulting in AFB_1_ in the raw material remaining in the pressed edible oil, resulting in AFB_1_ in the edible oil [5]. In China, incidents of AFB_1_ exceeding the standard in edible oil are not uncommon. Many unscrupulous traders and small oil extraction workshops use simple tools to extract rapeseed oil, peanut oil, etc. On the one hand, the environment is simple and crude, the craftsmanship is simple, and the weather is humid and hot. On the other hand, the lack of quality control treatment such as screening, alkali refining, and adsorption for oil plants is prone to the problem of AFB_1_ exceeding the standard [6]. Therefore, there is an urgent need to achieve rapid, batch, and accurate detection of AFB_1_ in edible oils.

In recent years, scientists and researchers from various countries have proposed many detection methods for AFB_1_ in food [7,8,9]. In China, “Limits of Mycotoxins in Foods” (i.e., GB 2761-2017) stipulates that the limit value of AFB_1_ in peanut oil and corn oil is 20 μg/kg, and the limit value of AFB_1_ in other vegetable oils and fats is 10 μg/kg [10]. “Determination of Aflatoxins Group B and Group G in Food” (i.e., GB 5009.22-2016) provides the methods for the determination of AFB_1_ in food [11], including isotope dilution liquid chromatography-tandem mass spectrometry, high performance liquid chromatography-pre-column derivatization, high performance liquid chromatography-post-column derivatization, etc. The abovementioned methods are all laboratory detection methods, and the sample pretreatment process is cumbersome, the detection time is long, and the cost is high, and it is difficult to meet the needs of on-site rapid detection. Therefore, it is particularly necessary to establish a rapid, green and accurate detection method for AFB_1_ in edible oil.

Raman spectroscopy uses inelastic scattering to analyze the sample, the sample does not need pretreatment, and the instrument is simple and convenient to operate [12]. In recent years, Raman spectroscopy combined with chemometric methods has been widely used in the fields of variety identification, quality analysis, and adulteration detection of edible oils [13,14,15,16,17,18]. However, in the detection of mycotoxins in edible oils [19], the existing research has used the synthesis of highly specific substrate materials to enhance the absorption intensity of specific Raman characteristic peaks. Then, a linear regression model between single or multiple characteristic peak variables and mycotoxins was established to realize the quantitative detection of mycotoxins in edible oil. For the existing research, on the one hand, the researchers need to have specific expertise in materials science and molecular science, and on the other hand, there is a high linear correlation between the response intensity of the characteristic peak and the mycotoxin content. However, the above two requirements are difficult to meet in actual field detection, especially the linear relationship between variables and target attributes. Therefore, surface-enhanced Raman spectroscopy can improve the detection limit of mycotoxins in laboratory studies. However, most of the existing research belongs to labeled detection, which not only has high requirements on researchers but also is difficult to meet the needs of modern rapid detection technology.

In view of this, this study started with Raman spectroscopy itself, starting from the preparation of peanut oil contaminated with aflatoxins, directly collecting Raman spectra of peanut oil samples without using any Raman enhancer. Then, the collected Raman spectra were denoised and characteristic wavelengths optimized from the point of view of numerical calculation using chemometric methods. In addition, chemometric models based on the optimized characteristic wavelength variables were established to realize the quantitative detection of AFB_1_ in peanut oil, so as to meet the needs of rapid quantitative detection of AFB_1_ in edible oil. Finally, the results obtained from our study were compared with those of existing studies using chemometric models constructed from theoretically calculated or empirically obtained Raman features.

## 2. Materials and Methods

### 2.1. Preparation of Peanut Oil Samples

First, 50 kg of white peanuts (i.e., peanut kernels of naturally dried) were purchased from a local supermarket, and the origin is Henan, China. The white peanut (*Arachis hypogaea* L.) used in the experiment was Kai Nong Bai No. 2. The purchased peanut kernels were then placed in an average of 4 moisture-absorbing cartons, inserting thermometers and hygrometers. In order to improve the process of peanut mildew, water mist was regularly sprayed every day to keep the humidity in the carton at about 85% and the temperature at 26–30 °C.

**Figure 1:**
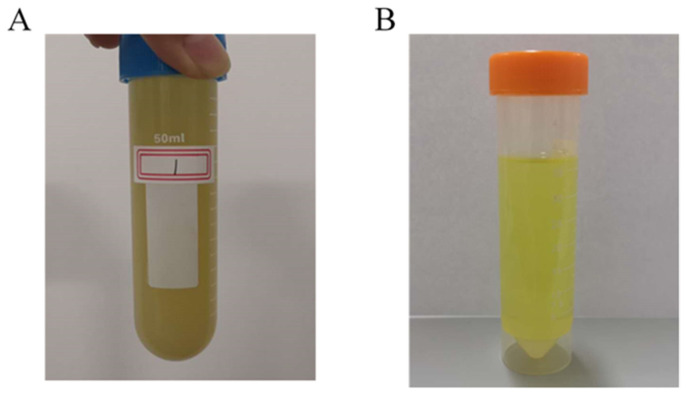
Crude oil (**A**) and crude oil after centrifugation (**B**) of peanut oil sample.

When preparing aflatoxin-contaminated peanut oil samples (50 mL), peanut samples (600 g) were taken from different positions in four cartons every other week, and the taken peanut samples were placed in a drying oven at 100 °C for 20 min. Then, the oil press was turned on and preheated for 30 min before oil extraction (the pressing raw peanut oil was shown in Figure 1A). In this study, the pressed raw peanut oil was subjected to three steps of filtration, precipitation and centrifugation. First, the pressed peanut oil was passed through a filter (stainless steel) screen to filter the oil residue. Next, the filtered peanut oil was left for 12 h, and the supernatant was taken to obtain the crude oil. Then the crude oil was put into a TGL-16M high-speed centrifuge (Hunan Xiangyi Centrifuge Instruments Co., Ltd., Changsha, China) and centrifuge at 11,000 r/min for 10 min to obtain the peanut oil samples (as shown in Figure 1B). Finally, the prepared peanut oil samples were stored in a refrigerator at 4 °C for future use.

### 2.2. Determination of Aflatoxin B_1_

The first method in GB 5009.22-2016, i.e., isotope dilution liquid chromatography-tandem mass spectrometry [11], was used to determine the AFB_1_ content in the prepared peanut oil samples. The detection process was roughly as follows: The AFB1 in the sample was first extracted with acetonitrile-water solution or methanol-water solution. Then, the extract was diluted with phosphate buffer solution containing 1% TritonX-100, and purified and enriched by immunoaffinity column. Finally, after the purification solution was concentrated, fixed to volume and filtered, it was separated by liquid chromatography, and quantified by tandem mass spectrometry detection and isotopic internal standard method.

### 2.3. Acquisition of Raman Spectra

In this study, the DXR laser Raman spectrometer (Thermo Fisher, Waltham, MA, USA) was used to collect the Raman spectra of the peanut oil samples, and the OMNIC software (Thermo Fisher, Waltham, MA, USA) was used to record the spectral data of the peanut oil samples. Before spectral acquisition, the instrument parameters were set as follows: The wavelength of the laser light source was 532 nm; the laser power was 10 mW; the focal length of the eyepiece was 10X; the integration time was 10 s, the spectral scanning range was 100–3300 cm^−1^, and the ambient temperature was controlled at about 20 °C.

**Figure 2:**
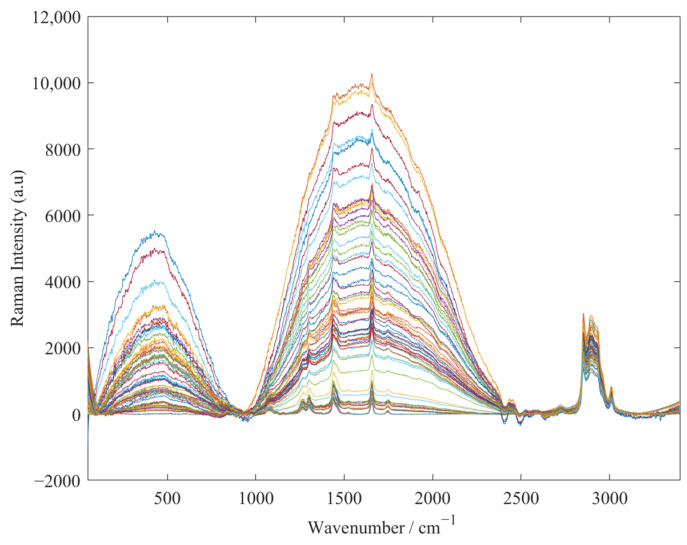
Raw Raman spectra of all peanut oil samples.

During the spectral acquisition process, 0.5 μL of peanut oil sample was drawn onto the silicon wafer by a pipette. Then, the silicon wafer was placed on the Raman spectrometer experimental platform for spectral collection of the peanut oil sample. The coarse adjustment knob of the Raman spectrometer was slowly adjusted to focus the objective on the sample. The OMNIC software was used to observe spectral imaging of the sample on the bench. Then, we fine-tuned the knob and performed the final spectral scan when the spectrum was stable and the noise was small. Since the spectral peak information in the range of 800–1800 cm^−1^ was rich and clear, the spectra in this wavelength range were studied as the original Raman spectra of peanut oil samples, as shown in Figure 2.

### 2.4. Spectral Pretreatment Methods

The raw spectra acquired from the Raman spectrometers usually contain background information and noise, which means that the raw spectral data must be preprocessed before model calibration; otherwise, it may cause large biases in subsequent data analysis. The spectral preprocessing methods used in this study were as follows:

(1) Wavelet transform (wavelet transform, WT). WT is a time and frequency manipulation of a class of signals. In the time and frequency domains, the effective signal is continuous, while the noise is discrete [20]. Therefore, after WT, the absolute value of the coefficient of the effective signal is larger, and the threshold method can be used to filter the signal. Since the shoulder widths of the Raman peaks are inconsistent, it is necessary to select appropriate wavelet parameters to achieve a good noise filtering effect. In this study, when using WT to process Raman spectra, the wavelet basis function selected the “sym5” function, and the decomposition level was set to 5. The Raman spectral signal was decomposed and reconstructed using the automatic denoising functions “wden” and “minimaxi” thresholds of the one-dimensional signal to remove the noise signal.
(2) Savitzky–Golay (SG) smoothing method. SG, also known as polynomial smoothing algorithm, was proposed by Savitzky and Golay and applied to data smoothing and noise filtering. When the SG preprocesses the spectral data, after defining the width of the window (which must be an odd number), a polynomial is used to fit the spectral variables in the window to predict the spectral intensity at the central wavelength point of the window [21]. As the window passes over the spectral data, the original value at the center of the window is replaced with the fitted value, resulting in smoothed data [22]. In this study, when processing Raman spectra with the SG, the polynomial order and the size of the smoothing window were set to 3 and 19, respectively.
(3) Adaptive iteratively reweighted penalized least squares (airPLS). The airPLS calculates the spectral baseline by iteratively adjusting the weights of penalized least squares [23]. In the iterative solution, the weight remains unchanged, and the algorithm makes an adaptive adjustment to the fidelity weight based on the difference between the spectrum and the fitting baseline of the previous iteration.

### 2.5. Variable Selection Method

Competitive adaptive reweighted sampling (CARS) is based on the jungle rule of survival of the fittest [24]. It uses an exponential decay function (EDF) combined with adaptive reweighted sampling (ARS) to select spectral variables with large absolute values of regression coefficients in the calibration model, while eliminating variables with small weights. During this process, cross-validation is used to evaluate the root mean square error of cross-validation (RMESCV) value of the subsets, and the subset with the lowest value is selected as the best subset.

In this study, CARS was used to optimize the Raman spectral features after preprocessing with 5-fold cross-validation. When CARS was running, the number of iterations was set to 5. Due to the randomness of the CARS algorithm, the study repeated running the CARS algorithm 50 times, and recorded the running results with the smallest RMSECV as the final screening wavelength variables.

### 2.6. Partial Least Square

Partial least squares (PLS) regression first linearly combines the original variables to obtain their principal components, that is, the number of PLS factors. Then, multiple linear regression is performed using the PLS factor as a new variable. The PLS has the advantage of being able to perform regression modeling even when the independent variables are linearly correlated, avoiding the defects of overfitting or insufficient use of spectral information [25].

Here, the PLS was applied to construct a regression model of the AFB_1_ content in peanut oil based on optimizing characteristic wavelength variables. During the model calibration process, a 5-fold cross-validation method was used to determine the optimal number of PLS factors.

### 2.7. Model Evaluation

In this study, the root mean square error of cross-validation (RMSECV) and the coefficient of correction determination ($R_{C}^{2}$) were used to evaluate the detection accuracy of different PLS models, and the root mean square error of prediction (RMSEP) and coefficient of predictive determination ($R_{P}^{2}$) to evaluate the generalization performance of different PLS models.

## 3. Results

### 3.1. Results of Spectral Pretreatment

**Figure 3:**
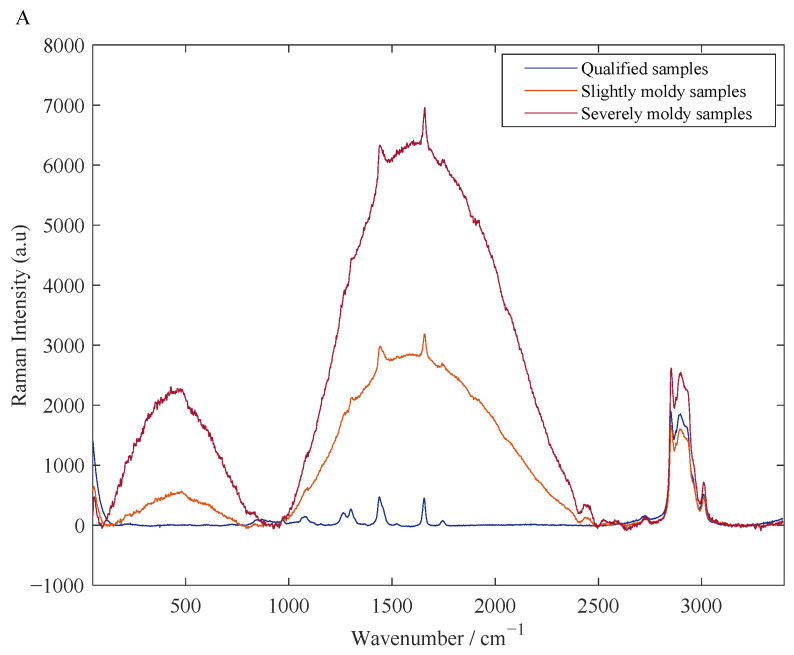

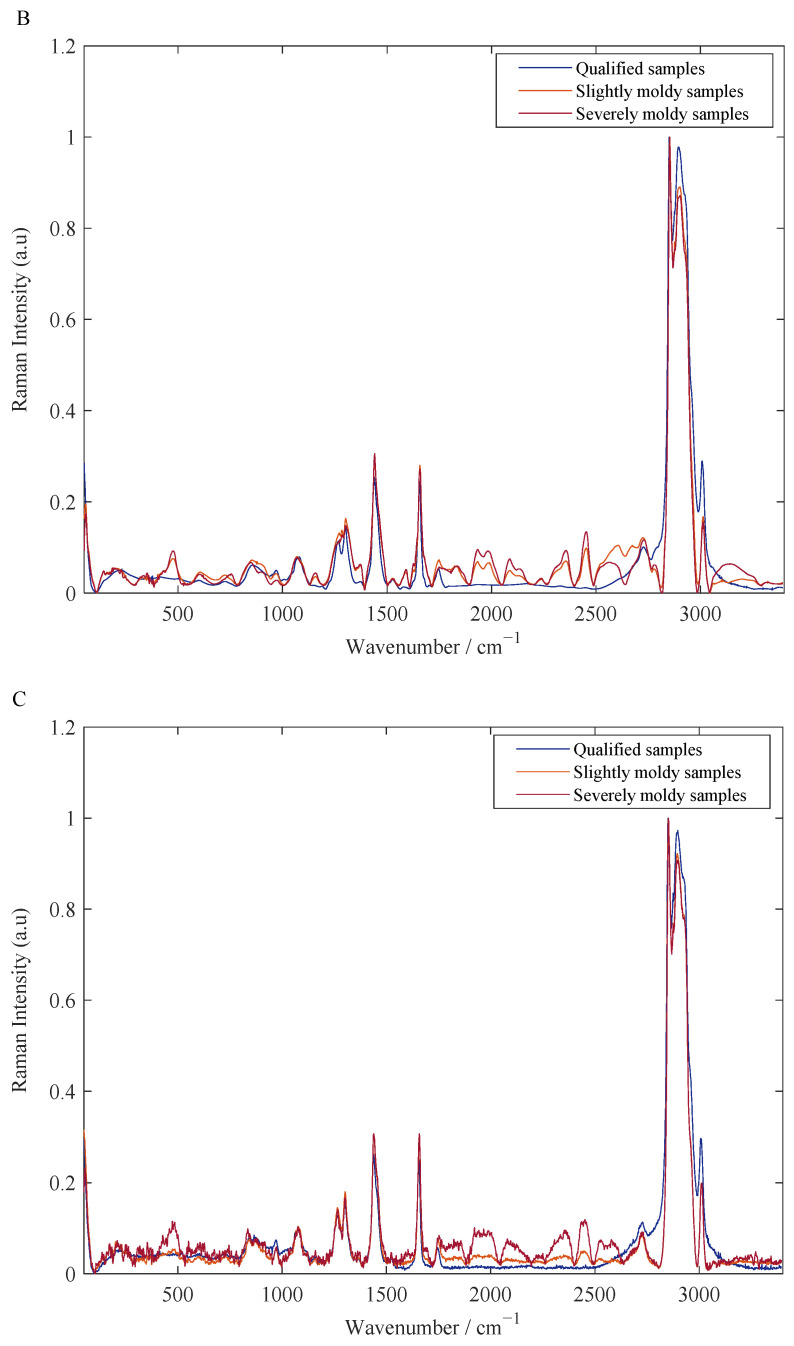

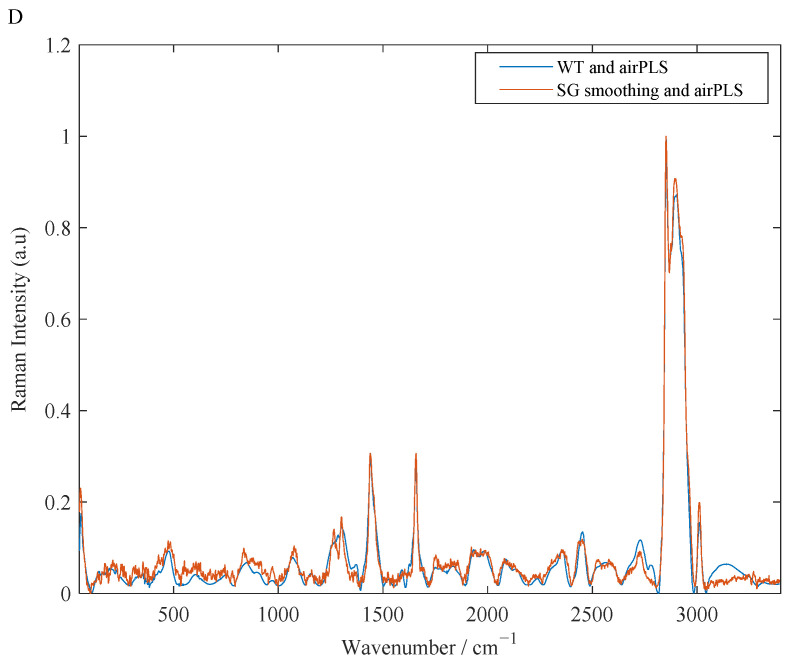
Raman spectra after pretreatment. (**A**) Raw Raman spectra of different degrees of mildew; (**B**) Raman spectra of different degrees of mildew after pretreatment with WT and airPLS; (**C**) Raman spectra of different degrees of mildew after SG smoothing and airPLS pretreatment; (**D**) Comparison of spectra of two preprocessing methods.

Figure 3A shows the raw Raman spectra of the collected peanut oil samples with different levels of aflatoxin contamination, including uncontaminated samples (AFB_1_ content below the national standard limit of 20 µg/kg) and mildly contaminated samples (AFB_1_ content below 20–100 µg/kg) and heavily contaminated samples (AFB_1_ content greater than 100 µg/kg). It can be seen from Figure 3A that with the increase of pollution degree, the content of AFB_1_ in peanut oil increases, and the baseline of the Raman spectrum of the sample gradually drifts, showing an “arch bridge” shape and becoming more and more obvious. This may be due to the increasingly strong fluorescence interference caused by the heavy contamination of the peanut oil with mold. The above facts indicate that the quantitative detection of AFB_1_ in peanut oil by Raman spectroscopy is feasible. In addition, the original Raman spectrum has great noise and baseline drift, which seriously affects the shape of the spectrum and will have a greater impact on the performance of the subsequent model building. Figure 3B shows the Raman spectrum after the WT and airPLS preprocessing and normalization. Compared with Figure 3A, after the WT–airPLS preprocessing, the phenomenon of noise burrs in the Raman spectrum is improved, the phenomenon of baseline drift is basically eliminated, and the signal characteristics are more obvious. Figure 3C is the Raman spectrum after the SG smoothing and airPLS preprocessing and normalization. It can be seen from Figure 3C that the burr phenomenon of the spectrogram after the SG smoothing still exists. That is to say, the SG smoothing does not completely eliminate the noise in the Raman spectrum with fluorescence effect, and the effect of eliminating noise is not as ideal as the preprocessing of the WT, which can be clearly seen in Figure 3D. In addition, comparing the effects of the airPLS combined with the SG smoothing and the WT respectively, we find that the Raman spectral baseline after preprocessing by the WT–airPLS is closer to 0. Therefore, we believe that the pretreatment method combined with WT–airPLS can effectively eliminate the irrelevant background information existing in the original Raman spectrum of peanut oil sample.

### 3.2. Division of Calibration Set and Prediction Set Samples

In order to ensure the rationality of the model results, the study divided the 80 peanut oil samples obtained in the experiment according to the following rules. First, the samples were reordered in ascending order of the AFB_1_ value. Then, in sorted order, the middle one of every five samples was taken into the prediction set, and the other four samples were entered into the calibration set. In this way, the calibration set had 64 peanut oil samples, and the prediction set included 30 peanut oil samples. Table 1 shows the statistical results of the AFB_1_ content in peanut oil samples in the two sample sets. As can be seen from Table 1, the mean and standard deviation of AFB_1_ in the peanut oil samples in the two sample sets are not significantly different, the minimum value in the prediction set is larger than the minimum value in the calibration set, and the maximum value in the prediction set is smaller than the maximum value in the calibration set. Therefore, the sample division of the calibration set and the prediction set in this study are reasonable.

### 3.3. Results of Feature Selection by the CARS Method

**Figure 4:**
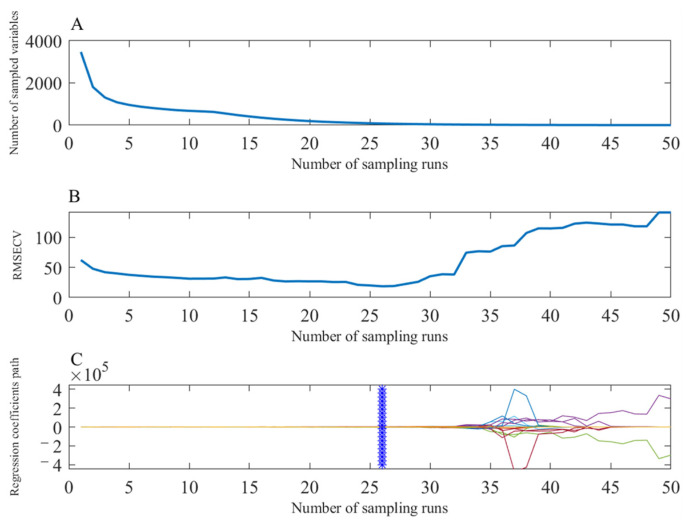
Results of the CARS method with the increasing of sampling runs. (**A**) the number of sampled variables; (**B**) RMSECV values; (**C**) the regression coefficients of each variables.

Figure 4 shows the results of the characteristic optimization of the preprocessed Raman spectra by the CARS method. Figure 4A demonstrates that as the number of sampling increases, the number of selected wavelength variables decreases rapidly in the initial stage, then decreases slowly and finally stabilizes. This shows that the CARS algorithm has two processes of rough selection and fine selection in the optimization process, which can improve the efficiency of the algorithm. Figure 4B shows that with the increase of sampling times, the RMSECV first gradually decreases to the lowest and then increases. This is because the RMSECV value decreases due to the elimination of a large amount of useless information at first, and then the RMSECV value increases due to the elimination of some useful information. Figure 4C shows the regression coefficients of the retained variables after each iteration of the CARS. The larger the coefficient, the more likely the corresponding variable is left behind, and the remaining variable is considered as the key variable. In this study, when the CARS iterated to 26 times, the RMSECV value of the PLS model established on the retained characteristic variables reached the minimum value, which was 18.6 μg/kg^−1^. At this time, the number of features retained by the CARS algorithm is 77, accounting for about 2.2% of the original spectral variables, which greatly reduces the spectral dimension and reduces the time and space complexity of subsequent modeling.

### 3.4. Results of the PLS Models Built on Optimized Features

**Figure 5:**
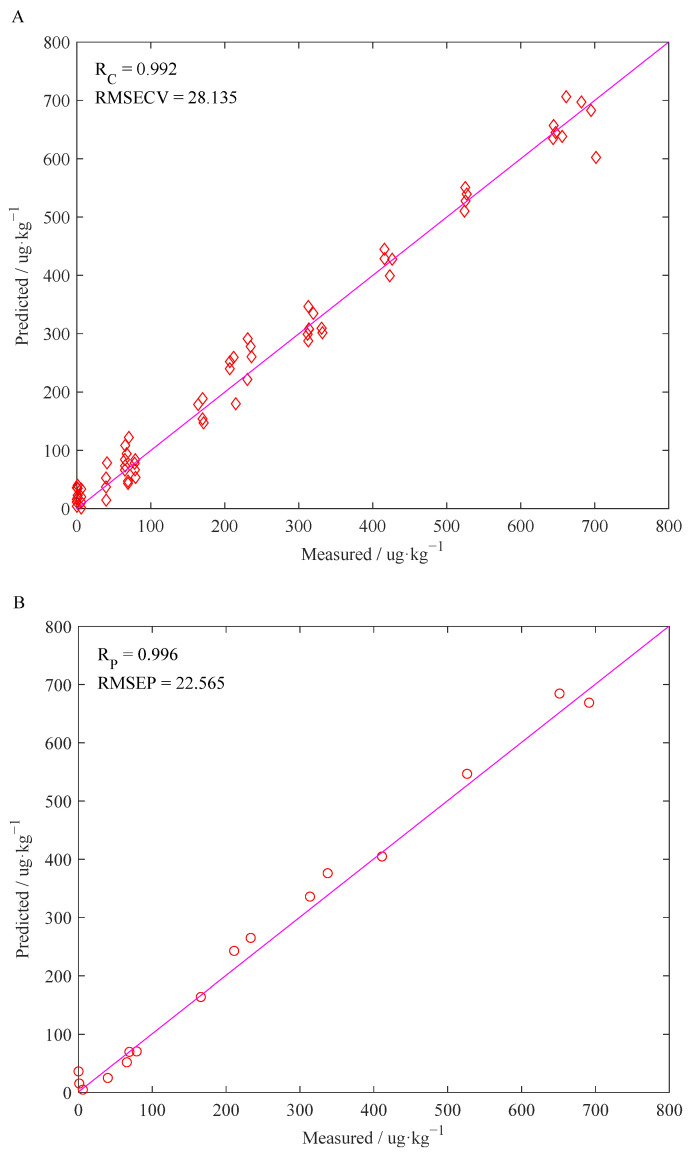
Comparison between the predicted value and the actual value. (**A**) Calibration set; (**B**) Prediction set.

This study used 77 Raman spectral features optimized by CARS to build a PLS model to realize the prediction of the AFB_1_ content in peanut oil. In the model calibration process, the optimal number of PLS factors was determined with the smallest RMSECV value. Figure 5 shows the scatter plot of the predicted values of the best PLS model based on 15 PLS factors and the measured values. The RMSECV, $R_{C}^{2}$, RMSEP, and $R_{P}^{2}$ of the model were 28.1 µg/kg, 0.98, 22.6 µg/kg, and =0.99. The results show that it is feasible to apply the Raman spectra combined with chemometrics to quantitatively detect AFB_1_ in peanut oil with high accuracy, and the established CARS–PLS model has high detection accuracy and nearly perfect generalization performance.

## 4. Discussion

To verify the importance of optimization of spectral feature variables, the performance of the CARS–PLS model was compared with that of a PLS model built on full spectral variables (FULL–PLS). In addition, in order to verify the hypothesis proposed at the beginning of this study, this study also established a PLS model (DFT–PLS) based on the Raman spectral characteristic peaks of the AFB_1_ proposed in the existing literature, and the performance of the model was compared with that of the CARS–PLS model. Among them, the selected characteristic peaks of the AFB_1_ are calculated based on density functional theory (DFT), and the specific characteristic peaks are listed in Table 2.

Table 3 shows the detection performance and generalization performance of different PLS models. As can be seen from Table 3, the PLS model established by the variables screened by CARS has the best detection accuracy and generalization performance, whether in the calibration set or in the prediction set. This shows that CARS is an effective Raman spectral feature wavelength selection method, which can eliminate the adverse effects of various non-target factors and obtain better prediction performance with fewer variables. In addition, for the FULL–PLS model, the number of spectral variables used to build the model is about 45 times that of the CARS–PLS model, but its $R_{P}^{2}$ is 11.2% lower, and its RMSEP is 70.6 ug/kg higher. For the DFT–PLS model, the $R_{P}^{2}$ of the model was 14.4% lower than that of the CARS–PLS model, while the RMSEP was 101.9 µg/kg higher. Among the three PLS models, the DFT–PLS model has the worst performance. This directly proves that the hypothesis put forward at the beginning of the study is correct. We can interpret the above results as follows:

(1) The absorption intensity of the Raman spectrum is related to the number of functional groups. As the degree of mildew increases, it causes a baseline shift in the Raman spectrum. And the more severe the mildew, the stronger the fluorescence effect is and the more serious the baseline drift is. That is to say, the increase of AFB_1_ content in peanut oil will cause corresponding changes in the Raman spectrum. Much existing literature also theoretically calculates and characterizes the Raman characteristic peaks of the AFB_1_, but they are all based on trace amounts of pure AFB_1_ or adding the pure product to non-mold edible oil. In this regard, we compared the theoretical characteristic peaks of the AFB_1_ calculated by the DFT with the Raman peaks collected by the actual experiment, and used these Raman peaks as input to construct the PLS model, but the model results were not satisfactory. This may be because in the process of mildewing of peanut oil in a high temperature and high humidity environment, the types of moldy fungi are not single (there may also be other molds, such as Salmonella, etc.), and this may result in the presented Raman peaks being a superposition of different mold contaminants, which is quite different from the theoretically calculated Raman spectrum of the AFB_1_. In addition, the Raman spectra of the same substance collected on different types of instruments will have deviations in the position of the spectral peaks and differences in the heights of the spectral peaks. Moreover, the surrounding environment of the AFB_1_ molecule in the experimental and theoretical calculations is also different. There is intermolecular interaction in the experiment, while the theoretical calculation simulates gas-phase molecules. Therefore, using the variable selection algorithm to optimize the collected spectral features from the perspective of numerical calculation can effectively reduce the spectral dimension and improve the accuracy and robustness of the subsequent model.
(2) The performance of the CARS–PLS model is better than that of the FULL–PLS model, which directly shows the importance of variable selection in the model calibration process. When Raman spectra are collected, they will be disturbed by environmental, human operation and instrumental factors, resulting in a large amount of useless and irrelevant information in the obtained spectra. The FULL–PLS model construction utilizes full-spectrum variables, and the large amount of useless and irrelevant information will directly affect the generalization performance and stability of the constructed detection model. Therefore, a more reliable detection model can be obtained by optimizing the characteristic wavelengths in the model calibration process.

Furthermore, the methods and results of our current study also have certain advantages compared to existing related studies. For example, Yang et al. assessed the feasibility of mid-infrared (MIR) technique to screen AFB1- and AFT-positive peanut oil. Different models could both reach 100% in calibration and validation [26]. However, the AFT-contaminated samples used in their study were produced using the general AFTs induction methods. This is essentially different from our current study, and their study is still at the stage of qualitative analysis. Chen et al. developed a laser-induced fluorescence spectroscopy (LIF) system for rapid and noninvasive screening of four varieties of peanut oils contaminated with different levels of AFB_1_. The feasibility of LIF technique for the rapid and nondestructive detection of AFB_1_ contamination in different varieties of edible oils was proved in this study [27]. However, the Yang et al. study only used different techniques, and the sample preparation was also artificially designed, and it was still at the stage of qualitative analysis. In addition, Chen et al. developed a surface-enhanced Raman scattering (SERS) aptasensor for ultrasensitive AFB_1_ detection using the amino-terminal AFB_1_ aptamer (NH2-DNA1) [28]. Their study, as we mentioned earlier, requires the synthesis of highly specific Raman enhancers to achieve the absorption intensity of the Raman characteristic peaks, thereby realizing the ultrasensitive detection of AFB_1_ content in peanut oil. However, their research has high requirements for the basic knowledge of chemistry possessed by the testers, and it is difficult to achieve large-scale promotion and application. In addition, the samples used in their study were not naturally AFB_1_-containing edible oil samples. On the contrary, the results obtained from our current study are more in line with actual production applications, and the field test is not too demanding for operators.

Although our current study has certain potential advantages compared with existing related studies, it also has certain limitations. For example, there is a single species of edible oil containing AFB_1_ in the current study. Only a single variety of peanut oil was included in the study. Therefore, in the follow-up study, we should further expand the type and quantity of the edible oil samples so as to improve the feasibility of practical application of the research results. In addition, the AFB_1_-containing peanut oil samples obtained in our current study were prepared by pressing moldy peanut raw materials. Due to the inappropriate control of the mildew process of peanuts during the experiment, the AFB_1_ content in some peanut oil samples obtained in the experiment exceeded the national standard too much, which is unlikely to occur in the actual production of edible oil. Therefore, this issue is also one of the key issues that our follow-up study needs to focus on.

## 5. Conclusions

The study verified the feasibility of Raman spectroscopy combined with multivariate analysis to achieve high-precision detection of AFB_1_ in the pressing peanut oil. This study started from the simulated peanut oil extraction experiment, and the Raman spectrometer was used to collect the Raman spectra of the peanut oil samples. The WT–SG and WT–airPLS were respectively pretreated on the raw Raman spectra of the peanut oil samples, and the pretreatment effects were compared. CARS was used for variable screening of the pretreated Raman spectra, and a PLS model based on optimized characteristic wavelengths was established to achieve high-precision detection of the AFB_1_ in peanut oil. The study can provide a technical basis for the research and development of portable Raman spectroscopy detection devices, and also provide a method reference for the effective law enforcement of quality supervision departments.

## Figures and Tables

**Table 1:** Statistical results of the AFB_1_ value of peanut oil samples in the calibration set and the prediction set.

Sample Sets	Sample Number	Maximum/μg·kg^−1^	Minimum/μg·kg^−1^	Mean/μg·kg^−1^	Standard Deviation/μg·kg^−1^
Calibration set	64	701.7	0.097	236.8	224.2
Prediction set	16	691.5	0.11	237.6	231.0

**Table 2:** Raman characteristic peak attribution.

Raman Spectra Calculated by DFT (cm^−1^)	Raman Spectra Collected Experimentally (cm^−1^)	Spectral Attribution
686	670	Ring breath(pyrane)
1076	1059	*v*(C-C-C)
1330	1267	*v*(C-O-C)
1393	1347	*v*(C-O)(ring skeleton vibration)
1603	1559	C-H def, *v*(C-C), *v*(C=C)
1645	1601	C-H def, *v*(C-C), *v*(C=C) (ring skeleton vibration)
1806	1701	*v*(C=O) (cyclopentene ring)
1883	1764	*v*(C=O) (pyrane ring)

**Table 3:** Prediction results of different PLS models.

Models	Number of Variables	Parameters	Calibration Set		Prediction Set	
			RMSECV/μg·kg^−1^	$R_{C}^{2}$	RMSEP/μg·kg^−1^	$R_{P}^{2}$
FULL–PLS	3468	PCs = 14	64.6	0.92	93.1	0.88
DFT–PLS	8	PCs = 5	107.7	0.67	124.5	0.73
CARS–PLS	77	PCs = 15	28.1	0.98	22.6	0.99

## Data Availability

The data are currently classified and will be available in 2024 with permission from the project.
